# Introducing *Pediatric Reports:* Only Another Pediatric Journal?

**DOI:** 10.3390/pediatric12030026

**Published:** 2020-11-16

**Authors:** Maurizio Aricò

**Affiliations:** Giovanni XXIII Children Hospital, Azienda Ospedaliero-Universitaria Consorziale Policlinico, 70126 Bari, Italy; maurizio.arico@policlinico.ba.it

The purpose of this editorial is to introduce Pediatric Reports, a 10-year-old journal that is now at a turning point. On 16 October 2020, MDPI took over from PAGEPress as the publisher of Pediatric Reports. Founded in 1996, MDPI (Multidisciplinary Digital Publishing Institute), an Open Access academic publisher, publishes 283 titles, including 78 Science Citation Index Expanded—Web of Science (Clarivate Analytics)—covered journals [1]. MDPI is the largest Open Access publisher and the fourth largest academic publisher in the world, publishing around 110,000 articles annually, of which 103,000 are research manuscripts and reviews [2].

## 1. Pediatric Reports: Which Mission?

Based on the above information, it is very clear that our journal has transitioned to a very prestigious international publisher. This offers the opportunity to move into a new phase of its development: towards a more global profile, with higher reputation, and possibly standing as a distinctive journal in the field of Pediatrics, with attention to emerging areas, practice-oriented approaches, and a relevant link between academic research and professional practice.

Nowadays, academic careers depend not only on the good content of published papers but also on the metrics of the indexes of the journal where the paper has been published (i.e., Thomson Reuters Impact Factor/InCites Journal Citation Reports–Clarivate; Scopus). Thus, we all should be devoted to gain the improvement of these indexes. We need to reach in the very near future two main targets: attribution of an Impact Factor, and a tremendous increase in the number of citations for at least the majority of published papers. We acknowledge being too “young” to receive definitive breakthrough papers. Yet, we definitely need to be smart enough to recognize, among the very many submitted manuscripts, those, which appear to be promising enough to be well cited by the peers. This either because they address new or interesting issues, or they make the right questions; those the readers are waiting for because of contemporary events, such as the current pandemic); those papers which contribute interesting, although preliminary, observations, or those papers we might be looking for when coping an unusual clinical case.

We may want to share with our potential performing authors an alliance: if you dare to publish your best article with us, we will make any effort to make this paper more and more recognized in the next years, also by a higher/official IF.

Once the research has been completed and the paper written, the legitimate expectation and desire of the authors is that it is going to be published as soon as possible. On our side, we feel engaged to provide a very quick turnaround in manuscripts handling. As of today, the average time from submission to publication across all MDPI journals is 39 days and the acceptance rate is 40% [2]. This has definitely been a strategy clue to MDPI’s success and escalation in the rating of the scientific publishers. How can we contribute to this effort? We will work to customize a rich and smart editorial board, to be continuously updated and enriched with fresh blood. Good advises and suggestions will always be taken very seriously, not only from the members of the board, but also from any reader or author. In keeping with the COPE code, we will ensure publication of negative results, which sometimes may be very helpful to direct new research hypotheses and strategies [3]. MDPI is not afraid of allowing enough space for publishing negative results, although they may be expected to be less cited [4]. “Studies that report negative studies should not be excluded”, says COPE, and we will keep this statement in our pocket.

Nobody can always be right, including the reviewers and the Editors. You may happen to have your paper not perfectly judged, by another journal or by Pediatric Reports. Writing to the Editor in Chief to comment on what you feel has been an inadequate or inaccurate review may sometimes offer a second opportunity to acceptable papers to get their space. If you feel strongly about it, just try it with us. We will consider such papers with an open mind.

Usually papers report the results of a research. Yet, you may happen sometimes to design a study or a trial that you feel is missing in the arena. In such situation, Pediatric Reports will be happy to consider submission of a study design paper.

As already remarked in other MDPI journals [5], there is much room to promote discussion about study results, and letters may be a useful and easy tool on this way. We plan to encourage the use of the Letter section to make the debate more lively and fast.

The choice of open access publishing has some good reasons. The main good reason is that your paper will be universally accessible to every reader, regardless of his/her access to resources allowing subscription to the journals. This will give the authors a good payback for their effort, with wider reading and, expectedly, more citing. On our side, MDPI is listed as a publisher in the Directory of Open Access Journals (DOAJ) (https://doaj.org/).

Finally, let us talk about the quality of the manuscript. We are fully aware that, although scientific English is universally used, non-mother tongue authors may often submit papers in which the language may be largely improved. We will take much care about the quality of your research and, even more, of the comments and interpretation you will deliver in the paper. The editorial office may help you to improve the language of your paper, but not the quality of your data or of your take-home message.

In summary, among the continuing benefits for Pediatric Reports’ authors, the Journal has and will continue to/will aim to improve to have:

- An unbiased peer-review, including internal review by Section Editors and external reviewers, experts on the topic;
- An international and subspecialty oriented diversity of the new and continuing members of the Editorial Board and reviewers;
- An editorial independence;
- Solid ethics principles, to avoid scientific or ethical misconduct.

## 2. The Editorial Board

Our Journal started publications in 2010 with the previous Publisher, but has not yet been able to reach its prestigious space in the arena. The present low calculated IF (÷ 0.45) with a continuing previous negative trend requires tremendous efforts to get an improvement. Similarly, the low Journal h-index of 16 and the SCImago Journal Rank (0.131, i.e., within the third quartile) also clearly need efforts to be improved. This effort has to be taken on by the Editor, together with the entire Editorial Board. Pediatric Reports needs to expand its editorial board, to reach new areas of research and at least some of the many pediatric subspecialties areas. An engaged and active Editorial Board is vital to make the journal grow. I am keen to work with an Editorial Board that is diverse and inclusive; I would therefore like to extend an invitation to active researchers to apply to join the Editorial Board of Pediatric Reports. The decision to appoint will be based on enthusiasm and motivation, and publication trajectory. Young, motivated and promising authors will be welcome, aside well-recognized investigators and clinicians.

Accountability must be part of our policy. Thus, to summarize our mission, the Journal is ready to:

- present annual analyses of performance and impact metrics, to check the expected positive trend and test how Pediatric Reports is performing versus competitors;
- expand its horizons and bring greater visibility by addressing hot and emerging hot topics;
- improve its scientific quality and international reputation, starting from today and over the next few years;
- improve publication speed and author communication;
- critically review the Journal acceptance and rejection rates to keep the quality high;
- be ready to increase the number of papers published, by acting on publication frequency, and number of pages per issue;
- accelerate the indexing in SCOPUS;
- establish collaborations with Scientific Societies and Associations in order to reach new interested and promising audiences;
- be ready to consider featuring special issues, allowing attracting new authors among which some already high-performing ones;
- promote Best Papers Awards.

Please, consider Pediatric Reports a good option for publication of your research papers. We will try to keep up with your expectation.
